# The wider societal benefits of surgical interventions for lymphatic filariasis morbidity management and disability prevention

**DOI:** 10.1371/journal.pntd.0009701

**Published:** 2021-09-16

**Authors:** Sarah Martindale, John Chiphwanya, Dorothy Emmie Matipula, Paul Ndhlovu, Hannah Betts, Louise A. Kelly-Hope

**Affiliations:** 1 Centre for Neglected Tropical Diseases, Department of Tropical Disease Biology, Liverpool School of Tropical Medicine, Liverpool, United Kingdom; 2 Ministry of Health, Lilongwe, Malawi; Washington University School of Medicine, UNITED STATES

## Introduction

Lymphatic filariasis (LF) is targeted for global elimination as a public health problem by interrupting transmission with mass drug administration and providing an essential package of care to people affected by the debilitating lymphedema and hydrocoele conditions [1]. In recent years, many LF endemic countries have scaled up their morbidity management and disability prevention (MMDP) programmes with a new focus on universal health coverage, primary healthcare strengthening, and integrated management of skin neglected tropical diseases (NTDs), with the aim of fully integrating quality services for LF MMDP into national health systems to ensure sustainability [2].

The positive impact of MMDP interventions for patients has been documented [3–6]; however, no research has been conducted on the wider societal benefits, including the impact on the people who care for patients, i.e., caregivers. Ton and colleagues [7] calculated that the burden of depressive illness in LF patient caregivers was 229,537 disability-adjusted life years (DALYs). Other studies have documented a negative socioeconomic impact on the caregivers of people affected by filarial and nonfilarial leg lymphedema and those who experience painful secondary bacterial infections, acute adenolymphangitis [8–10].

Hydrocoele is the most common LF clinical manifestation, which affects approximately 19 million men worldwide and can be cured by surgery [11]. In Malawi, recent large-scale patient mapping and modelling estimate that at least 14,000 men have hydrocoele across the country. In 2015, surgical campaigns were initiated to address the burden, together with a study to highlight the significant positive impact of surgery on men in highly endemic areas [4].

We advocate that the positive impact of this surgical intervention can extend beyond the patient to include their caregivers, who are likely to be family members (predominately female) and have their own time, work, and quality of life affected.

## Evidence from the field

### Ethics statement

Ethical approval was obtained from the Malawi National Health Sciences Research Committee (Protocol 15/3/1406) and the Liverpool School of Tropical Medicine Research Ethics Committee (Protocol 15.047). All adult participants provided written informed consent; no children participated in the study.

### Methods

To provide evidence of the wider societal benefits of surgical intervention, we extended the study in Malawi by Betts and colleagues [4] to include a small retrospective survey on caregivers, 6 months postsurgical intervention, to better understand their (i) characteristics and relationship to the hydrocoele patient; (ii) level of assistance provided: number and type of physical activities they assisted with; (iii) impact on time and work: number of days per month they provided care and took off work; (iv) impact on the quality of life in relation to their usual activities (3 questions), social issues (3 questions), and psychological health (4 questions) using a similar scale-based scoring system, adapted from the Lymphatic Filariasis Quality of Life Questionnaire (LFSQQ) [12,13] to quantify problems, i.e., no problem = 0, mild = 1, moderate = 2, and severe = 3, with individual total scores ranging from 0 = no problem to 30 = severe problem in all 10 questions. Average scores for each question across the 3 domains were also calculated. All questions were asked twice by a trained field research team member: first for the caregiver to recall the situation presurgery when the patient had his hydrocoele and second to state the situation postsurgery after the patient’s recovery. Caregivers were also asked if they provided additional care during the recovery period immediately after surgery, the number of days they provided additional care, and time taken off work during the recovery period.

### Results

#### Caregiver characteristics

We surveyed 40 randomly selected caregivers. The majority were female (75%); median age 36 years (female 39 years; male 27 years); approximately half had no schooling and were illiterate (55%), and one-third (35%) had primary school level education. Most caregivers were wives (65%) of the patient. Other female caregivers included daughters (*n* = 2), a granddaughter (*n* = 1), and a sister (*n* = 1). Male caregivers included brothers (*n* = 3), fathers (*n* = 2), sons (*n* = 3), a grandson, (*n* = 1), and a cousin (*n* = 1), thus highlighting the wide involvement of the extended family.

#### Assisted activities

The number of physical activities the caregivers assisted the patient with pre- and postsurgery is summarised in Table 1. Presurgery, 11 (27.5%) caregivers reported that they provided no additional physical help on a specific activity for the patient, which increased nearly 3-fold postsurgery to 31 (77.5%). Presurgery, 17 (42.5%) caregivers assisted with 3 or more activities to patients, which decreased postsurgery to 5 (12.5%) caregivers.

**Table 1:** Level of assistance caregivers provided to patients pre- and postsurgery.

Number of activities assisted	Presurgery		Postsurgery	
	*n*	%	*n*	%
No assistance needed	11	27.5%	31	77.5%
1 activity	5	12.5%	2	5.0%
2 activities	7	17.5%	2	5.0%
3 activities	5	12.5%	2	5.0%
4 activities	3	7.5%	1	2.5%
5 activities	6	15.0%	0	0.0%
6 activities	2	5.0%	2	5.0%
7 activities	1	2.5%	0	0.0%
**Total**	40		40	

**Fig 1:**
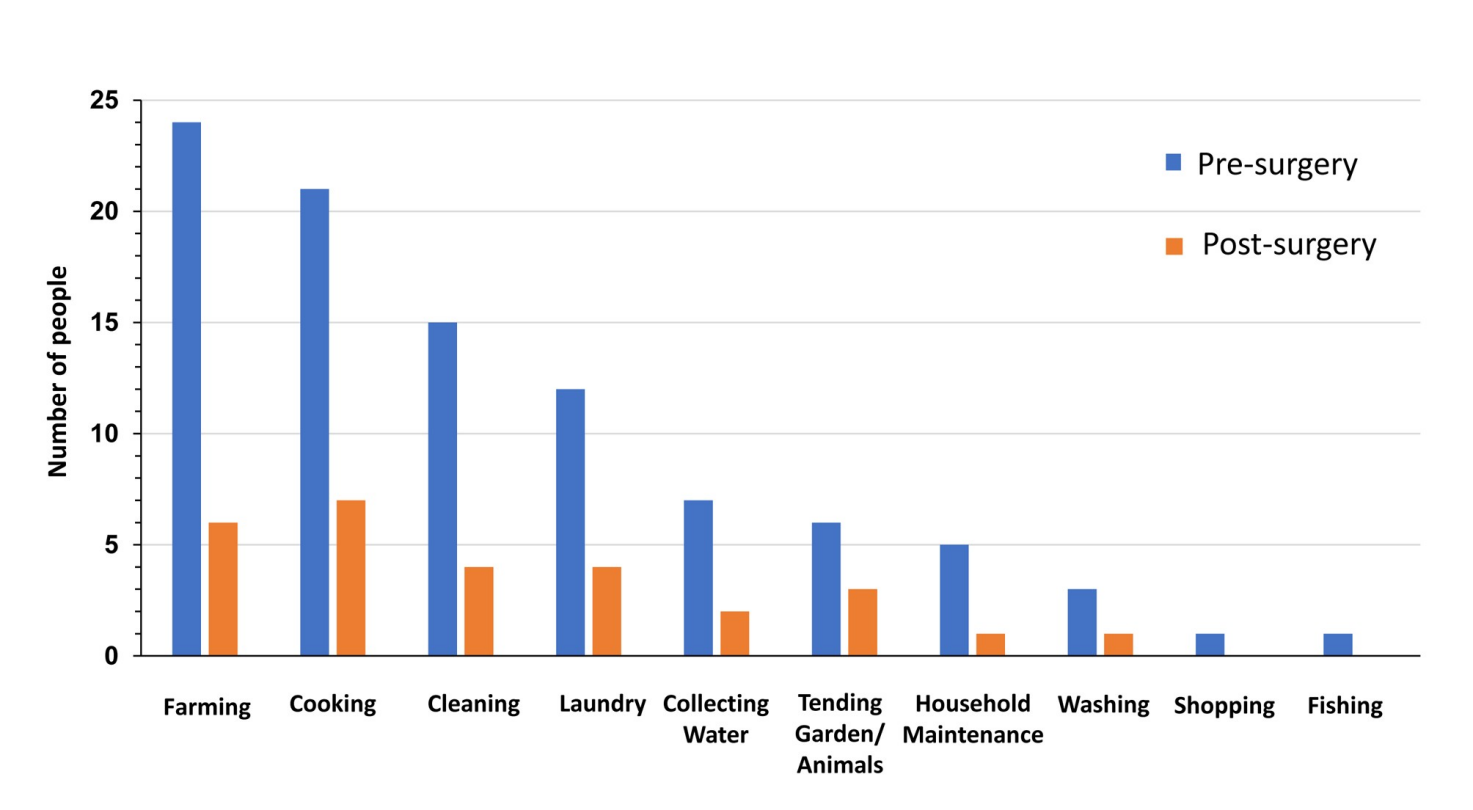
The types of activities caregivers assisted the patient with pre- and postsurgery.

Presurgery, the activities that caregivers (*n* = 29; 72.5%) physically assisted with most frequently included farming (*n* = 24; 82.8%), cooking (*n* = 21; 72.4%), cleaning (*n* = 15; 51.7%), laundry (*n* = 12; 41.4%), and least frequently shopping and fishing (*n* = 1; 3.4%). When considering male caregivers only who provided care presurgery (*n* = 8; 80%), the majority reported assisting with farming (*n* = 6; 75.0%) followed by cooking (*n* = 4; 50%), suggesting that male caregivers were not more likely to assist with certain activities, compared with female caregivers. However, given the small sample size, further data are needed to understand the implications of gender on caregiving responsibilities. Postsurgery, across all tasks, fewer caregivers stated that they provided assistance with each specific task as shown in Fig 1.

#### Impact on time and work

Presurgery, caregivers (*n* = 29) assisted the patient with physical activities for an average of 25.3 days per month or a total of 733.0 caregiver days. Postsurgery, only 5 caregivers continued to assist with activities for an average of 26.2 days per month or a total of 131.0 caregiver days. Presurgery, the caregivers were unable to go to work due to caring responsibilities for an average was 8.2 days per month. Postsurgery, no caregivers took time off work to assist the patient. During the initial recovery period postsurgery, 20 caregivers provided additional help. This recovery period lasted an average of 30.8 days postsurgery, with caregivers providing care for an average of 24.7 days or a total of 494 caregiver days. During this recovery period, 18 (90%) of the 20 caregivers were unable to work for an average of 13.7 days.

#### Quality of life

**Fig 2:**
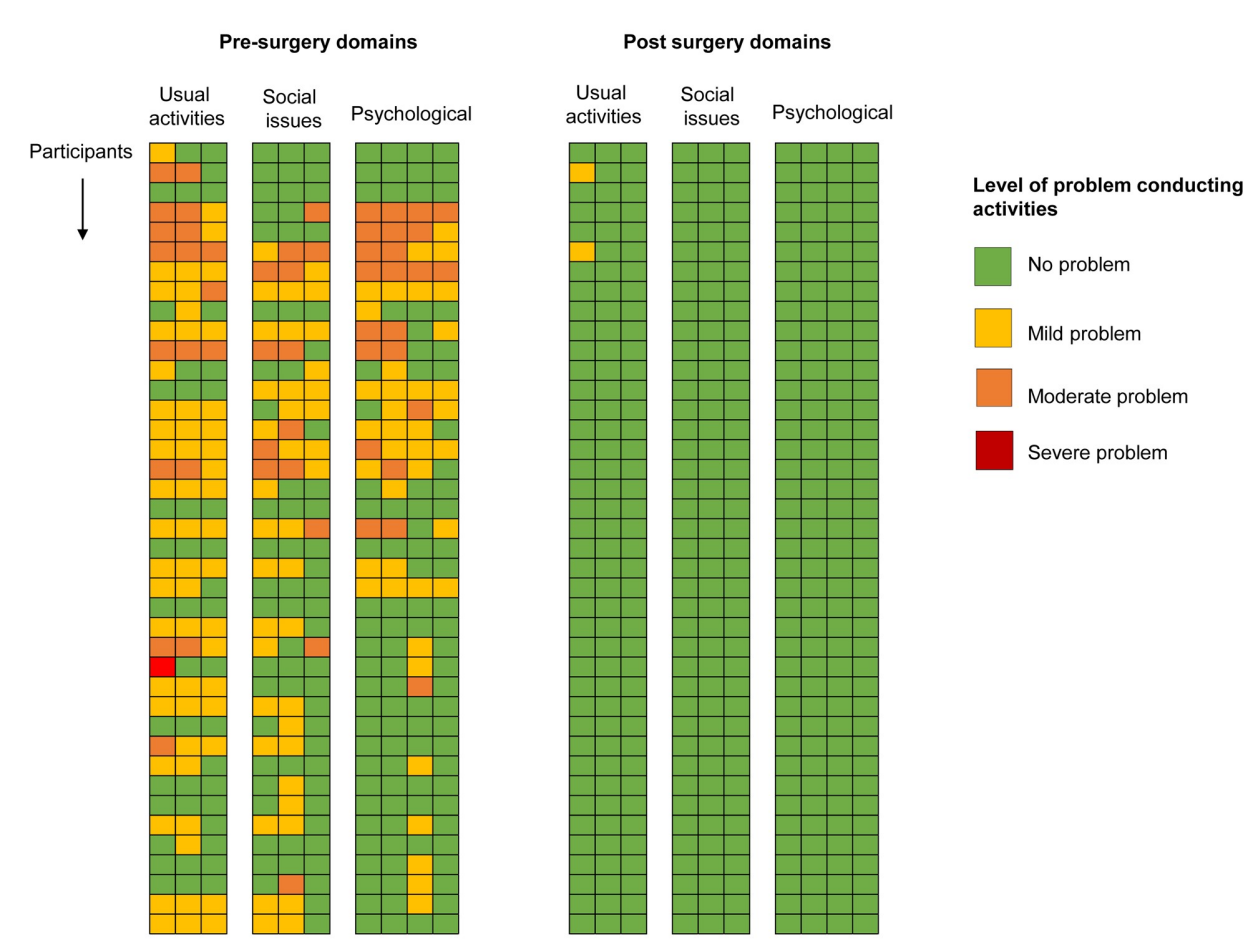
Quality of life responses across 3 domains colour coded by the level of problem caregivers’ experiences presurgery and postsurgery.

Presurgery, the majority of caregivers reported mild, moderate, or severe problems when supporting the hydrocoele patients, with individual overall quality of life scores ranging from 0 to 17. Average scores for questions on usual activities ranged 0.6 to 0.95, social issues 0.4 to 0.575, and psychological health 0.325 to 0.625. A summary of presurgery proportions of problem level for each question is below and included in S1 Table. Postsurgery, all caregivers, except 2 (question on their own job), reported that they no longer had problems, with scores decreasing significantly to 0 (paired *t* test; *p* < 0.05). The significant changes pre- and postsurgery are visually highlighted in colour coded Fig 2 and differences in average scores in S1 Table.

### Presurgery summaries

Usual activities: Did helping cause problems in doing your (1) own job (mild 47.5%, moderate 20%, and severe 2.5% problem), (2) own household activities (mild 50% and moderate 17.5%), and (3) usual leisure activities (mild 45% and moderate 7.5%)?
Social issues: Did you have a problem with (1) joining in regular social activities outside your home (mild 37.5% and moderate 10%), (2) inside your home (mild 40% and moderate 15%), or (3) your romantic relationships (mild 20% and moderate 10%)?
Psychological health: Did you feel (1) worried about your own health (mild 17.5% and moderate 20%), (2) your future (mild 22.5% and moderate 20%), (3) neglected by friends and family members (mild 35% and moderate 12.5%), and (4) unable to make plans for the future (mild 22.5% and moderate 5%)?

#### Quotes

Caregivers provided additional comments on the positive changes to their lives following the patient’s surgery:

He is able to work, and we are working together to help the home (wife, 45 years).

He helps with household work now (wife, 21 years).

He is able to take care of me as a husband now (wife, 24 years).

Now, he is able to satisfy me sexually (wife, 36 years).

Now, we are able to face the community with no shame since my father is well (daughter, 23 years).

My friends stopped laughing at me (son, 19 years).

I am no longer worried about the future (wife, 36 years).

## Conclusions

These preliminary data on caregivers highlight the wider burden of filariasis morbidity on families and endemic communities if left untreated. Like the struggles that patients face before interventions, caregivers have their daily living activities, own time, and ability to work negatively affected by providing additional assistance on a range of tasks in and around the home. Further, there was a negative psychosocial impact, with caregivers’ friendships and intimate relationships thwarted, and many felt neglected, shamed, and concerned for the future [7,14].

Importantly, these preliminary data highlight the significant positive change that a surgical intervention can have on many aspects of caregivers’ lives, especially female family members. This has additional wide-ranging societal and economic benefits. It helps to address gender inequalities, which have been highlighted with lymphedema [10], and are vital to making progress towards the Sustainable Development Goal 5, in recognising and valuing unpaid care and domestic work [15]. It also suggests that the high benefit–cost ratio of 24.5 of surgery recently calculated for hydrocoele patients by Sawers and colleagues [16] could potentially be doubled if caregivers’ postsurgery economic productivity was included in the calculations. This highlights the value of this intervention, and a clear message of “buy 1 surgery and get 2 people back to work” may convince international donors and ministries of health to continue to invest. For example, in Malawi, this would mean that at least 28,000 people would benefit from 14,000 surgeries.

Therefore, we advocate that the continued support and scale-up of LF MMDP activities will have comprehensive physical, social, psychological, and economic benefits to patients, their families, and the endemic communities in which they live. This will further help national elimination programmes address the requirements of universal healthcare as part of WHO NTD Road Map, help families and communities get out of the poverty cycle, and ensure that no one is left behind [1].

## Supporting information

S1 Table Summary of the frequency, percentage, and mean of domain scores of the 40 caregivers pre- and postsurgery. (DOCX)

## References

[1] World Health Organization. Ending the neglect to attain the sustainable development goals: A road map for neglected tropical diseases. 2020. p. 2021–30.

[2] World Health Organization. Lymphatic filariasis—managing morbidity and preventing disability: an aide-mémoire for national programme managers [Internet]. 2nd ed. Geneva; 2021.

[3] Sawers L, Stillwaggon E. Economic Costs and Benefits of Community-Based Lymphedema-Management Programs for Lymphatic Filariasis in India. Am J Trop Med Hyg. 2020;103(1):295–302. Epub 2020 Jul 13. doi: 10.4269/ajtmh.19-0898 PubMed Central PMCID: PMC7356420. 32653050 PMC7356420

[4] Betts H, Martindale S, Chiphwanya J, Mkwanda SZ, Matipula DE, Ndhlovu P, et al. Significant improvement in quality of life following surgery for hydrocoele caused by lymphatic filariasis in Malawi: A prospective cohort study. PLoS Negl Trop Dis. 2020;14(5):e0008314. Epub 2020 May 10. doi: 10.1371/journal.pntd.0008314 PubMed Central PMCID: PMC7239494. 32384094 PMC7239494

[5] Douglass J, Mableson H, Martindale S, Jhara ST, Karim MJ, Rahman MM, et al. Effect of an Enhanced Self-Care Protocol on Lymphedema Status among People Affected by Moderate to Severe Lower-Limb Lymphedema in Bangladesh, a Cluster Randomized Controlled Trial. J Clin Med. 2020;9(8). Epub 2020 Aug 6. doi: 10.3390/jcm9082444 PubMed Central PMCID: PMC7464742. 32751676 PMC7464742

[6] Thomas G, Richards FO Jr, Eigege A, Dakum NK, Azzuwut MP, Sarki J, et al. A pilot program of mass surgery weeks for treatment of hydrocele due to lymphatic filariasis in central Nigeria. Am J Trop Med Hyg. 2009;80(3):447–51. Epub 2009 Mar 10. . 19270297

[7] Ton TG, Mackenzie C, Molyneux DH. The burden of mental health in lymphatic filariasis. Infect Dis Poverty. 2015;4:34. Epub 2015 Aug 1. doi: 10.1186/s40249-015-0068-7 PubMed Central PMCID: PMC4520254. 26229599 PMC4520254

[8] Martindale S, Mkwanda SZ, Smith E, Molyneux D, Stanton MC, Kelly-Hope LA. Quantifying the physical and socio-economic burden of filarial lymphoedema in Chikwawa District, Malawi. Trans R Soc Trop Med Hyg. 2014;108(12):759–67. doi: 10.1093/trstmh/tru154 . 25270880

[9] Caprioli T, Martindale S, Mengiste A, Assefa D, F HK, Tamiru M, et al. Quantifying the socio-economic impact of leg lymphoedema on patient caregivers in a lymphatic filariasis and podoconiosis co-endemic district of Ethiopia. PLoS Negl Trop Dis. 2020;14(3):e0008058. Epub 2020 Mar 4. doi: 10.1371/journal.pntd.0008058 PubMed Central PMCID: PMC7069637. 32126081 PMC7069637

[10] Martindale S, Mackenzie C, Mkwanda SZ, Smith E, Stanton MC, Molyneux D, et al. “Unseen” Caregivers: The disproportionate gender balance and role of females in the home-based care of lymphatic filariasis patients in Malawi. Front Womens Health. 2017;2(2):1–3. doi: 10.15761/FWH.1000126

[11] World Health Organization. Surgical approaches to the urogenital manifestations of lymphatic filariasis. Report from an informal consultation among experts. 2019 Contract No.: (WHO/CDS/NTD/PCT/2019.04).

[12] Aggithaya MG, Narahari SR, Vayalil S, Shefuvan M, Jacob NK, Sushma KV. Self care integrative treatment demonstrated in rural community setting improves health related quality of life of lymphatic filariasis patients in endemic villages. Acta Trop. 2013;126(3):198–204. Epub 2013 Mar 19. doi: 10.1016/j.actatropica.2013.02.022 23499714

[13] Stanton MC, Yamauchi M, Mkwanda SZ, Ndhlovu P, Matipula DE, Mackenzie C, et al. Measuring the physical and economic impact of filarial lymphoedema in Chikwawa district, Malawi: a case-control study. Infect Dis Poverty. 2017;6(1):28. Epub 2017 Apr 4. doi: 10.1186/s40249-017-0241-2 PubMed Central PMCID: PMC5376674. 28366168 PMC5376674

[14] Litt E, Baker MC, Molyneux D. Neglected tropical diseases and mental health: a perspective on comorbidity. Trends Parasitol. 2012;28(5):195–201. Epub 2012 Apr 6. doi: 10.1016/j.pt.2012.03.001 . 22475459

[15] The United Nations General Assembly. Transforming our World: The 2030 Agenda for Sustainable Development. New York; 2015.

[16] Sawers L, Stillwaggon E, Chiphwanya J, Mkwanda SZ, Betts H, Martindale S, et al. Economic benefits and costs of surgery for filarial hydrocele in Malawi. PLoS Negl Trop Dis. 2020;14(3):e0008003. Epub 2020 Mar 27. doi: 10.1371/journal.pntd.0008003 PubMed Central PMCID: PMC7094819. 32210436 PMC7094819

