# Supplementary material for: The wider societal benefits of surgical interventions for lymphatic filariasis morbidity management and disability prevention
Source: PLoS Negl Trop Dis. 2021 Sep 16;15(9):e0009701. doi: 10.1371/journal.pntd.0009701 (PMC8445426; doi:10.1371/journal.pntd.0009701)
Supplement: S1 Table — (DOCX) [file pntd.0009701.s001.docx]

Supplementary file

| **Domain** | **Before/After Surgery** | **Question ^a^** | **No Problem (0)** | | **Mild Problem (1)** | | **Moderate Problem (2)** | | **Severe Problem (3)** | | **Mean Score** | **Mean Difference [95% CI]^c^** |
| --- | --- | --- | --- | --- | --- | --- | --- | --- | --- | --- | --- | --- |
|  |  |  | **N** | **%** | **N** | **%** | **N** | **%** | **N** | **%** |  |  |
| Usual Activities | Before | 19. Their own job | 12 | 30% | 19 | 48% | 8 | 20% | 1 | 3% | 0.95 | 0.90 ^b^  [0.64, 1.16] |
|  | After |  | 38 | 95% | 2 | 5% | 0 | 0% | 0 | 0% | 0.05 |  |
|  | Before | 20. Household activities | 13 | 33% | 20 | 50% | 7 | 18% | 0 | 0% | 0.85 | 0.85 ^b^  [0.63, 1.07] |
|  | After |  | 40 | 100% | 0 | 0% | 0 | 0% | 0 | 0% | 0.00 |  |
|  | Before | 21. Usual leisure activities | 19 | 48% | 18 | 45% | 3 | 8% | 0 | 0% | 0.60 | 0.60 ^b^  [0.40, 0.80] |
|  | After |  | 40 | 100% | 0 | 0% | 0 | 0% | 0 | 0% | 0.00 |  |
| Social Issues | Before | 22. Social issues outside the home | 21 | 53% | 15 | 38% | 4 | 10% | 0 | 0% | 0.58 | 0.58 ^b^  [0.36, 0.79] |
|  | After |  | 40 | 100% | 0 | 0% | 0 | 0% | 0 | 0% | 0.00 |  |
|  | Before | 23. Social issues inside the home | 18 | 45% | 16 | 40% | 6 | 15% | 0 | 0% | 0.70 | 0.70 ^b^  [0.47, 0.93] |
|  | After |  | 40 | 100% | 0 | 0% | 0 | 0% | 0 | 0% | 0.00 |  |
|  | Before | 24. Personal relationships | 28 | 70% | 8 | 20% | 4 | 10% | 0 | 0% | 0.40 | 0.40 ^b^  [0.19, 0.61] |
|  | After |  | 40 | 100% | 0 | 0% | 0 | 0% | 0 | 0% | 0.00 |  |
| Psychological Health | Before | 25. Worried about own health | 25 | 63% | 7 | 18% | 8 | 20% | 0 | 0% | 0.58 | 0.58 ^b^  [0.32, 0.83] |
|  | After |  | 40 | 100% | 0 | 0% | 0 | 0% | 0 | 0% | 0.00 |  |
|  | Before | 26. Worried about future | 23 | 58% | 9 | 23% | 8 | 20% | 0 | 0% | 0.63 | 0.63 ^b^  [0.37, 0.88] |
|  | After |  | 40 | 100% | 0 | 0% | 0 | 0% | 0 | 0% | 0.00 |  |
|  | Before | 27. Neglected by friends and family | 21 | 53% | 14 | 35% | 5 | 13% | 0 | 0% | 0.60 | 0.60 ^b^  [0.38, 0.82] |
|  | After |  | 40 | 100% | 0 | 0% | 0 | 0% | 0 | 0% | 0.00 |  |
|  | Before | 28. Unable to make plans for the future | 29 | 73% | 9 | 23% | 2 | 5% | 0 | 0% | 0.33 | 0.33 ^b^  [0.15, 0.51] |
|  | After |  | 40 | 100% | 0 | 0% | 0 | 0% | 0 | 0% | 0.00 |  |
| ^a^ Questions focussed on the impact of providing care on aspects of the caregivers lived: “Did helping the person affected with hydrocoele…..”  ^b^ Significant (p<0.05)  ^c^ Equal variances assumed | | | | | | | | | | | |  |
